# Sex and gender medicine in physician clinical training: results of a large, single-center survey

**DOI:** 10.1186/s13293-016-0096-4

**Published:** 2016-10-14

**Authors:** Shivani Dhawan, May Bakir, Erika Jones, Sarah Kilpatrick, C. Noel Bairey Merz

**Affiliations:** 1 Barbra Streisand Women’s Heart Center, Cedars-Sinai Heart Institute, Los Angeles, CA USA; 2 Department of Obstetrics and Gynecology, Cedars-Sinai Medical Center, Los Angeles, CA USA

**Keywords:** Sex, Gender, Physician, Training

## Abstract

**Background:**

“Sex and Gender Medicine” is a novel medical discipline that takes into account the effects of sex and gender on the health of women and men. The Institute of Medicine in the USA declared in its 2001 and 2010 statements that being a woman or a man significantly impacts the course of diseases, and therefore, this fact must be considered in diagnosis and therapy. We evaluated the representation of Sex and Gender Medicine in clinical training at Cedars-Sinai Medical Center, a large, tertiary, non-profit, academic medical training center in the Western United States.

**Methods:**

Post-graduate physician trainees (residents and fellows) in all medical and surgical departments (medicine, surgery, OB-GYN, pediatrics, anesthesiology, pathology, urology, electrophysiology, pulmonary critical care, cardiology, women’s heart, medical genetics, radiology, neurosurgery, and radiation oncology) were surveyed online; 80 (55 and 45 % female and male residents, respectively) out of 890 physicians (9 % response rate) responded to questions regarding sex and gender-based medicine.

**Results:**

Seventy percent of post-graduate physician trainees indicated that gender medicine concepts are never or only sometimes discussed/presented in their training program. Slightly greater than 70 % of the trainees indicated that gender concepts are never or only sometimes incorporated into didactic lectures or clinical teaching. However, more than 65 % felt that gender medicine concepts are important, and 60 % agreed that gender medicine curriculum should be implemented and taught in their clinical program.

**Conclusions:**

Current physician trainees endorse both a current lack of and need for Sex and Gender Medicine clinical training.

**Electronic supplementary material:**

The online version of this article (doi:10.1186/s13293-016-0096-4) contains supplementary material, which is available to authorized users.

## Background

“Sex and Gender Medicine” is a novel medical discipline that takes into account the effects of sex and gender on the health of women and men [1]. The Institute of Medicine in the USA declared in its 2001 and 2010 statements that being a woman or a man significantly impacts the course of diseases, and therefore, this fact must be considered in diagnosis and therapy [2].

We evaluated the representation of Sex and Gender Medicine in clinical training at Cedars-Sinai Medical Center, a large, tertiary, non-profit, academic medical training center in the Western United States.

## Methods

Post-graduate physician trainees (residents and fellows) in all medical and surgical departments (medicine, surgery, OB-GYN, pediatrics, anesthesiology, pathology, urology, cardiology-electrophysiology, pulmonary critical care, cardiology, women’s heart, medical genetics, radiology, neurosurgery, and radiation oncology) (*n* = 890) were surveyed online. The survey link was distributed in February, 2014, to physician trainees via Cedars-Sinai email and re-distributed once a week for an additional 4 weeks with reminder emails. The last participant completed the survey 45 days from the survey launch. The survey questions used the term “Gender Medicine” rather than “Sex and Gender Medicine” to avoid internet filters and are shown in Additional file 1. Eighty of the 890 (9 %) including 55 % female and 45 % male trainees and PGY levels 1 (24 %), 2 (16 %), 3 (22 %), 4 (11 %), 5 (8 %), 6 (5 %), and 7 (14 %) completed the survey. Responses were tallied and presented as percentages of respondents.

## Results

**Fig. 1:**
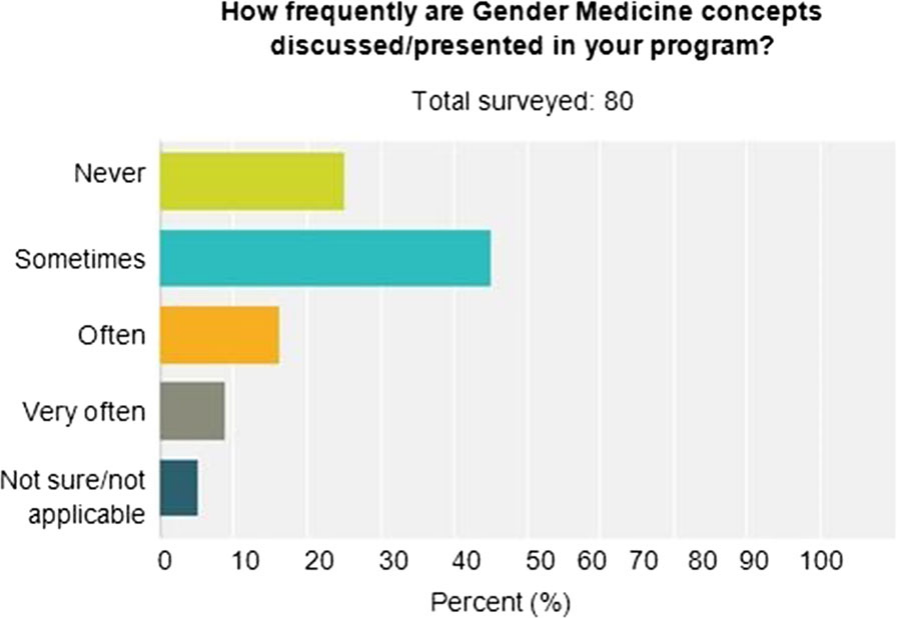
How frequently are gender medicine concepts discussed/presented in the program

**Fig. 2:**
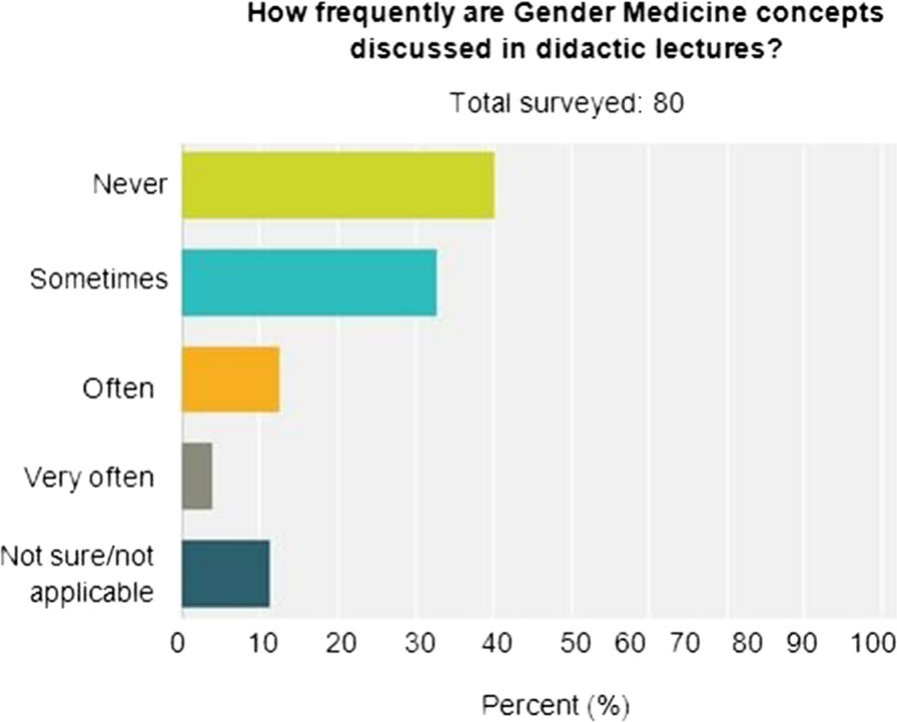
How frequently are gender medicine concepts discussed in didactic lectures

**Fig. 3:**
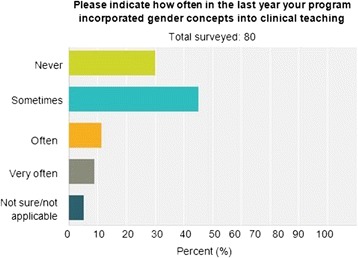
How often is gender medicine included in clinical teaching

The number and percentage of each specialty that answered the survey are represented in Table 1. More than 65 % of post-graduate physician trainees felt that sex and gender medicine concepts are important, and 60 % agreed that sex and gender medicine curriculum should be implemented and taught in their clinical program. However, 70 % indicated that sex and gender medicine concepts are never or only sometimes discussed/presented in their training program (Fig. 1), and slightly greater than 70 % indicated that gender concepts are never or only sometimes incorporated into didactic lectures (Fig. 2) or clinical teaching (Fig. 3). Less than 10 % of trainees have never taken gender into account when treating a patient. The survey results differed by physician’s sex for three of the survey questions. More than 80 % of female physician trainees felt that sex and gender medicine concepts are important and were never or only sometimes discussed during clinical training program compared to less than 65 % of male physician trainees. Over 95 % of female physicians indicated sometimes, often, or very often taking gender into account when treating a patient compared to only 65 % of male physician trainees. Furthermore, 17 % of male physician trainees reported never taking gender into account when treating a patient whereas there were no female physician trainees who reported never taking gender into account when treating a patient.

**Table 1:** Percentage of physicians by specialty

Department	Percentage
Anesthesiology (7)	10.96
Cardiology (2)	2.74
Electrophysiology (1)	1.37
Genetics (1)	1.37
Internal medicine (20)	27.40
Neurology (1)	1.37
Neurosurgery (2)	2.74
OB-GYN (3)	4.11
Pathology (9)	12.33
Pediatrics (12)	16.44
Pulmonary critical care (1)	1.37
Radiation oncology (2)	2.74
Surgery (8)	10.96
Urology (2)	2.74
Women’s heart (1)	1.37
Total	100.00

## Discussion

A majority of post-graduate physician trainees surveyed believe that sex and gender medicine concepts are important and should be implemented into their training program. Our survey was representative of many clinical disciplines, including women and men trainees, as well as medicine and surgical trainees of all PGY levels. Physician trainees also provided examples of past lecture titles which have incorporated sex and gender concepts (question 4 in Additional file 1). Examples included “Autism and Gender,” “Pain Management,” “Cardiac Disease in Women,” and “Differential Diagnosis Based on Gender.” These findings endorse that a majority of trainees have a basic understanding of sex and gender medicine concepts and would accept “Sex and Gender Medicine” incorporated into their clinical training.

Our survey results demonstrated that trainees endorse a current lack of sex and gender medicine concepts presented in their program, discussed in didactic lectures, or incorporated into clinical teaching. Indeed, a lack of attention to sex and gender differences in diseases which impact both women and men has increasingly been identified as contributing to health disparities, particularly in women [2]. Most prior reports regarding gender in medicine evaluate relations between the gender of the physician and outcome [3, 4] with essentially no evaluations regarding outcomes relative to physician training and sex and gender medicine concepts. One prior training report evaluated cultural competency although this did not specify gender [5]. Research testing differing educational strategies to optimize outcomes with regard to sex and gender-based medicine concepts for female and male patients is needed.

Notably, female physicians in training expressed that sex and gender medicine was important more often than their male counterparts. The women surveyed almost always took gender into account when treating patients, compared to the men surveyed (65 %). These findings may reflect “male standardization” of healthcare, whereby practice standards and guidelines of health conditions common to women and men typically reflect a male standard. Further work is needed to validate this potential sex difference among physicians and, if present, how it affects healthcare outcomes.

Notably, despite the lack of sex and gender-based medicine inclusion or integration in the clinical teaching program, the majority of physician trainees surveyed do take the gender of a patient into consideration when treating a patient. Indeed, the most feasible and relevant aspect of “personalized medicine” is taking the gender of the patient into account. Increasing attention to sex and gender-based medicine concepts throughout the medical training is needed to improve the health of women and men.

### Limitations

Due to the low response rate of the physician trainees, the results may not accurately represent all the opinions of the medical center’s physician trainee population. Further, our sample was underpowered to evaluate responses by medical specialty.

## Conclusions

Greater efforts are needed to implement sex and gender-based medicine concepts into training programs in didactic lectures and in clinical teaching.

## Additional file

Additional file 1: Gender Medicine Curriculum Survey. (PDF 223 kb)

## References

[1] Nobelius AM, J W: Gender and medicine: a conceptual guide for medical educators. Traralgon: Monash University School of Rural Health; 2004.

[2] Wizemann TM, Pardue M: Exploring the biological contributions to human health: does sex matter? Washington DC: The National Academies Press; 2001. 25057540

[3] Roter DL, Hall JA (2004). Physician gender and patient-centered communication: a critical review of empirical research. Annu Rev Public Health.

[4] Thom DH, Tirado MD, Woon TL, McBride MR (2006). Development and evaluation of a cultural competency training curriculum. BMC Med Educ.

[5] Roter DL, Hall JA, Aoki Y (2002). Physician gender effects in medical communication: a meta-analytic review. JAMA.

