# Supplementary material for: Sex and gender medicine in physician clinical training: results of a large, single-center survey
Source: Biol Sex Differ. 2016 Oct 14;7(Suppl 1):37. doi: 10.1186/s13293-016-0096-4 (PMC5073858; doi:10.1186/s13293-016-0096-4)
Supplement: Additional file 1: — Gender Medicine Curriculum Survey. (PDF 223 kb) [file 13293_2016_96_MOESM1_ESM.pdf]

APPENDIX

Gender Medicine Curriculum

* 1. Gender Medicine is a novel medical discipline that takes into account the effects of sex and gender on the health of women and men. The Institute of Medicine in the USA declared in its 2001 and 2010 statements that being a woman or being a man significantly influences the course of diseases and therefore this fact must be considered in diagnosis and therapy.

On a scale of 1 to 7, with 1 being extremely unimportant and 7 being extremely important, please rate how important you think gender medicine concepts are.

- ◯ 1
- ◯ 2
- ◯ 3
- ◯ 4
- ◯ 5
- ◯ 6
- ◯ 7

* **2. Please indicate how frequently gender medicine concepts are discussed/presented in your program.**

- ◯ Never
- ◯ Sometimes
- ◯ Often
- ◯ Very often
- ◯ Not sure/not applicable

*** 3. Please indicate how often in the last year your program incorporated gender concepts in didactic lectures**

- ◯ Never
- ◯ Sometimes
- ◯ Often
- ◯ Very often
- ◯ Not sure / not applicable

4. Give an example of a lecture title. If answered never in previous question please leave blank

[ ]

*** 5. Please indicate how often in the last year your program incorporated gender concepts into clinical teaching**

- ◯ Never
- ◯ Sometimes
- ◯ Often
- ◯ Very often
- ◯ Not sure / not applicable

*** 6. To what extent do you agree or disagree with the following statement: A Gender Medicine curriculum should be implemented and taught in your training program**

- ◯ Strongly Disagree
- ◯ Disagree
- ◯ Agree
- ◯ Strongly Agree
- ◯ Not Sure/not applicable

*** 7. Please estimate the number of times you have taken gender into account when treating a patient**

- Never
- Sometimes
- Often
- Very often
- Not sure / not applicable

*** 8. Please indicate your PGY level**

- 1
- 2
- 3
- 4
- 5
- 6
- 7

*** 9. Please indicate your department**

*** 10. What is your gender?**

- Female
- Male
